# A Blueprint for the Conduct of Large, Multisite Trials in Telemedicine

**DOI:** 10.2196/29511

**Published:** 2021-09-20

**Authors:** Patricia Commiskey, April W Armstrong, Tumaini R Coker, Earl Ray Dorsey, John C Fortney, Kenneth J Gaines, Brittany M Gibbons, Huong Q Nguyen, Daisy R Singla, Eva Szigethy, Elizabeth A Krupinski

**Affiliations:** 1 Division of Stroke Department of Neurology Vanderbilt University Medical Center Nashville, TN United States; 2 Department of Dermatology Keck School of Medicine University of Southern California Los Angeles, CA United States; 3 Department of Pediatrics University of Washington School of Medicine Seattle, WA United States; 4 Seattle Children's Research Institute Seattle, WA United States; 5 Center for Health + Technology University of Rochester Medical Center Rochester, NY United States; 6 Division of Population Health Psychiatry & Behavioral Sciences University of Washington Seattle, WA United States; 7 Center of Innovation for Veteran-Centered and Value-Driven Care Health Services Research and Development Department of Veteran's Affairs Seattle, WA United States; 8 Keck School of Medicine University of Southern California Los Angeles, CA United States; 9 Division of Health Services Research & Implementation Science Kaiser Permanente Southern California Pasadena, CA United States; 10 Center of Addiction and Mental Health Toronto, ON Canada; 11 Lunenfeld Tanenbaum Research Institute Toronto, ON Canada; 12 Department of Psychiatry University of Toronto Toronto, ON Canada; 13 Center for High Value Health Care, UPMC Insurances Division Department of Psychiatry, Medicine, and Pediatrics University of Pittsburgh Pittsburgh, PA United States; 14 Department of Radiology and Imaging Sciences Emory University School of Medicine Atlanta, GA United States

**Keywords:** telemedicine trials, randomized trials, challenges, multisite, mobile phone

## Abstract

Recent literature supports the efficacy and efficiency of telemedicine in improving various health outcomes despite the wide variability in results. Understanding site-specific issues in the implementation of telemedicine trials for broader replication and generalizability of results is needed. Lessons can be learned from existing trials, and a blueprint can guide researchers to conduct these challenging studies using telemedicine more efficiently and effectively. This viewpoint presents relevant challenges and solutions for conducting multisite telemedicine trials using 7 ongoing and completed studies funded by the Patient-Centered Outcomes Research Institute portfolio of large multisite trials to highlight the challenges in implementing telemedicine trials. Critical issues of ensuring leadership and buy-in, appropriate funding, and diverse and representative trials are identified and described, as well as challenges related to clinical, informatics, regulatory, legal, quality, and billing. The lessons learned from these studies were used to create a blueprint of key aspects to consider for the design and implementation of multisite telemedicine trials.

## Introduction

Multisite clinical trials are critical to advance the detection, diagnosis, and treatment of disease and to drive valid and reliable knowledge that can be generalized to broad integrated populations and health care settings. Techniques to conduct multisite clinical trials vary as a function of many factors, including the intervention under consideration, patient population, institutional setting, country, and so on, and a vast amount of literature exists on how to conduct clinical trials of all sorts. Telemedicine technologies and trials provide researchers not only the opportunity to assess the impact of telemedicine interventions in different populations but also to use these technologies in the evaluation of other clinical interventions where it is not the telemedicine aspect being evaluated.

Why do we need a blueprint for researchers conducting multisite telemedicine trials? A key reason is that telemedicine trials present new challenges to researchers that are typically not encountered in more traditional clinical trials. Despite the steady growth in acceptance and use, telemedicine is still far from being mainstream, and despite the creation of practice guidelines by the American Telemedicine Association and a variety of professional societies [1], there is little uniformity in practice and thus outcomes. Although existing literature supports the efficacy and efficiency of telemedicine in improving outcomes as measured using a variety of metrics, there is wide variability in these results. An extensive body of literature on modern telemedicine already exists, going back well over 25 years, with at least two mainstream peer review telemedicine journals dedicated to presenting research results with an increasing emphasis on outcomes. Systematic literature reviews and meta-analyses are being conducted at accelerating rates, which could not happen reliably if a large enough body of published studies to draw from were not available. However, this body of literature as a whole includes many mixed, inconclusive, and even contradictory results [2-6].

This really is not surprising. Medical research literature as a whole is often characterized as having mixed or inconclusive evidence, especially when, like telemedicine, the topic of interest is a new technology, procedure, treatment, or intervention. When a new tool or technology is introduced, others jump on board with their (often proprietary) versions, and isolated studies at single institutions are conducted on different patient and provider populations, with slightly (or very) different protocols, using different workflows, different metrics, benchmarks, and statistical analyses. In addition, the clinical application of telemedicine in medical and behavioral care rapidly expanded during the COVID-19 emergency state, allowing providers to reach many patients virtually because of relaxation of the Centers for Medicare & Medicaid Services regulations and state licensures and stay at home orders around the country [7-9]. Although there are many case reports of successes, large-scale systematic studies evaluating the effectiveness of telemedicine across different populations and settings are needed more than ever [10].

What can we do to get “better evidence in telemedicine”? As with any new medical intervention, we need more valid and reliable evidence to conduct large, multisite telemedicine trials. We need to understand and control (or at least account for) site-specific issues in implementation for broader replication and generalizability of the results. We need to acknowledge and understand the heterogeneity of treatment effects in diverse populations, and when examining the compendium of results, account for this heterogeneity appropriately before claiming a given telemedicine intervention does or does not *work*.

Given this opportunity and the difficulties of conducting multisite trials in telemedicine described in this paper, a blueprint can point the way for researchers to conduct these challenging studies more efficiently and effectively. This will build an evidence base for understanding the true impact and use of telemedicine.

## Multisite Telemedicine Clinical Trials

This paper examines 7 ongoing and completed studies funded by the Patient-Centered Outcomes Research Institute (PCORI) portfolio of large multisite trials to highlight the challenges in implementing telemedicine trials; each trial is described in Table 1. Key issues (eg, clinical, informatics, regulatory, legal, quality, and billing) are summarized and examples of how they were addressed are described, thereby providing a blueprint of topics that will typically need to be addressed in the planning and implementation of telemedicine clinical trials.

PCORI has funded more than 25 large, multisite, pragmatic trials in telemedicine, most of which are conducted across multiple states, with some in more than 10 different states. More than three-quarters of them are ongoing and at various stages of implementation. Representative trials were selected based on the stage of implementation and discussion with PCORI on which current trial participants might be willing to share their experiences.

**Table 1:** Selected large multisite trials funded by PCORI^a^.

Principal investigator	Study title	Summary	PCORI contract number	ClinicalTrials.gov number
AWA	Improving Specialty-Care Delivery in Chronic Skin Diseases	This study evaluated an innovative CCH^b^ model where patients and primary care providers could access dermatologists on the web directly and asynchronously via a pragmatic RCT^c^ to test whether a CCH model results in equivalent improvements in disease severity, quality of life, and mental health, and whether the model provides better access to specialty care, compared with usual in-person care for psoriasis management.	IHS-071502-IC	NCT02358135
TRC	Using Telehealth to Deliver Developmental, Behavioral, and Mental Health Services in Primary Care Settings for Children in Underserved Areas	Using community-engaged research principles, FQHC^d^ (n=6 sites) and CHMCs^e^ (n=2 sites) designed a telehealth-based intervention to improve the referral system for children being referred to specialty mental health care from primary care. We conducted an RCT involving 342 children, aged 5-12 years, with mental health concerns to receive either a routine referral or the new telehealth-enabled referral system.	IH-12-11-4168-IC	NCT02396576
ERD	Connect.Parkinson	Connect.Parkinson assessed the feasibility, value, and benefits of telemedicine visits with a specialist for individuals with Parkinson disease (n=200) through randomization to usual care or their routine care enhanced by video visits with a Parkinson specialist in approximately 20 US states.	AD-12-11-4701	NCT02038959
KJG	C3FIT^f^ Stroke Care Trial	C3FIT uses team-based, enhanced collaboration to follow patients from presentation at the Emergency Department through 12 months post discharge to compare joint commission-certified stroke care with an Integrated Stroke Practice Unit care model that uses nurse and lay health educator care teams to visit patients and caregivers at home or in rehabilitation or skilled nursing facilities to assess function and quality of life using telehealth technology for patients at 18 US clinical sites.	PCS-2017C3-9081	NCT04000971
HQN, Mularski RA	Noninferiority Comparative Effectiveness Trial of Home-Based Palliative Care (HomePal) Trial	HomePal is a CER^g^ comparing two models of home-based palliative care, standard approach with nurses and physicians making home visits versus nurses in patients’ home facilitating remote physician consults.	PLC-1609-36108	NCT03694431
DRS	SUMMIT^h^ Trial	SUMMIT examines whether psychotherapy delivered via telemedicine is as effective as in-person sessions for perinatal populations in Toronto, Canada; Chapel Hill, North Carolina; and Chicago, Illinois.	PCS-2018C1-10621	NCT04153864
ES	Specialty Medical Homes to Improve Outcomes for Patients With IBD^i^ and Behavioral Health Conditions Trial	This trial compares traditional face-to-face delivered to telemedicine delivered team-based care within a subspecialty medical home integrating behavioral and medical care for patients with IBD in Pittsburgh, Pennsylvania, New York City, New York, and Cleveland, Ohio.	IHS-2017C3-8930	NCT03985800

^a^PCORI: Patient-Centered Outcomes Research Institute.

^b^CCH: Collaborative Connected Health.

^c^RCT: randomized controlled trial.

^d^FQHC: federally qualified health center.

^e^CHMC: community mental health clinic.

^f^C3FIT: Co-ordinated, Collaborative, Comprehensive, Family-based, Integrated, Technology-Enabled Care.

^g^CER: community-engaged research.

^h^SUMMIT: Scaling Up Maternal Mental Healthcare by Increasing Access to Treatments.

^i^IBD: inflammatory bowel disease.

## Issues and Challenges in Implementing Multisite Telemedicine Clinical Trials

As researchers consider moving to multisite clinical trials using telemedicine, several issues need to be considered. Telemedicine has shown success in overcoming geographic and time limitations in medical care delivery, and evidence shows improved access to care, higher patient satisfaction, and enhanced quality of care and value of care can result [11]. Using telemedicine as a means of conducting a multisite clinical trial or using multiple sites to assess telemedicine intervention also raises some important decisions and considerations that should be addressed during study design and implementation. Textbox 1 summarizes the specific study elements and key considerations for assessing the impact of telemedicine interventions and the use of telemedicine to conduct multisite trials that were derived from reviewing completed and ongoing PCORI trials.

**Study elements and key considerations for assessing the impact of telemedicine interventions.**

**Technology**

- Selecting a telemedicine platform
- Information technology reviews and approvals
- Technology support for electronic failure and vendor changes
- Potential financial cost to patients and ways to mitigate

**Regulatory and reimbursement**

- Credentialing and privileging
- Insurance empaneling
- Health Insurance Portability and Accountability Act or security standards
- Billing and remuneration
- Contracting and budgeting
- Institutional review boards

**Design and initiation**

- Comparators and outcomes
- Cross-contamination
- Clinical workflow
- Patient and participant characteristics
- Training
- Health disparities

**Implementation and sustainability**

- Buy-in
- Patients and family caregiver engagement
- Sustainability

## Technology

### Overview

Selecting a telemedicine platform is a key first step in ensuring the success of the research project and should be carefully considered. In some cases, the research project will simply use the telehealth platform in which the institution or institutions are already using clinically. If a platform is not available, selecting one is a key aspect of study planning and execution and typically must be done in conjunction with the health care system. Considering the current availability, study context, cost, security, and compliance are essential. Furthermore, considering user-centeredness ensures that the study platform is acceptable and feasible for both the provider and target population. For example, the SUMMIT (Scaling Up Maternal Mental Healthcare by Increasing Access to Treatments) trial [12] used specific criteria to facilitate the selection of telemedicine platforms, including simplicity of use for patients and providers, the availability of easy-to-follow instructions, whether Health Insurance Portability and Accountability Act (HIPAA) or Personal Health Information Protection Act guidelines were met, and technical support. Finally, integration of the telemedicine platform with an institution’s electronic medical record (EMR) was an important consideration. A robust telemedicine platform may not always integrate with the institution’s EMR, which can present challenges in study visits, clinical care documentation, and continuity of care. In this case, using two EMR systems (one for institutional EMR and the other for telemedicine EMR) can be used, but the study protocol must specify the integration of both systems and procedures for reconciling differences to ensure that documentation is complete in both systems. Understanding the impact of using multiple systems is essential, and differences in the integration of the telemedicine platform across different study sites need to be evaluated in confounder analyses. The SUMMIT trial used an existing platform that was free and readily used by all registered health professionals in Toronto, Canada, but an existing platform was not available in Chicago, Illinois; therefore, the study team opted for a commercial product. In all cases, the platform offered a version that was compliant with either the United States HIPAA security rule or the Canadian Personal Health Information Protection Act. User-centeredness requires ensuring a platform that is acceptable and feasible for the provider and target population. The SUMMIT trial used specific criteria to facilitate telemedicine platform implementation, including simplicity of use with easy instructions, ability to try out the platform, and technical support.

### Information Technology Reviews and Approval

Researchers must work closely with their institution’s information technology (IT) department (university, hospital, or both) during evaluation and selection to ensure that telemedicine platforms meet federal, state, and institutional privacy and security standards and provide robustness for efficient patient-provider communication. As research requirements and approvals vary, involving institutional IT representatives early is critical. The study team may need to take the lead in evaluating telemedicine platforms, involving IT to understand the *must-have* standards, including encryption, data security, confidentiality, digital certificates, log-in IDs and passwords, auto-log-offs, audit trails, and disaster recovery plans. The Co-ordinated, Collaborative, Comprehensive, Family-based, Integrated, Technology-enabled Care (C3FIT) trial added a high-level IT leader at their main institution to the trial, providing a concrete effort for an IT liaison for all study years. Furthermore, this person was involved in design well before applying for funding, which allowed the IT leader to be fully vested in the study, providing both consultation and guidance through IT review and while setting up agreements with sites, as well as throughout the study to assist with technology changes and user issues. The importance of this as the COVID-19 pandemic expanded cannot be overstated. All of the trials worked closely with their IT teams for both technology selection and deployment, such as holding dedicated workshops to help train providers and patients, providing training materials either on the internet or through written materials, sending IT support to patient homes if necessary, or helping them remotely and often training providers to troubleshoot issues to some degree.

### Technology Support for Electronic Failure and Vendor Changes

Ensuring institutional support is essential both for the technology and to ensure seamless integration [13] into existing appointment, scheduling, documentation, panel management, and billing systems. Avoiding workarounds specific to research requires advance planning and ample time for reviews, approvals, and coordination across multiple business groups. Electronic failure and poor signal quality are unavoidable, especially in remote areas [14]. Contingency plans must be in place, for instance, to quickly switch from video to phone when connectivity is not reliable to avert unnecessary frustrations. Having service line agreements in place will ensure that IT is highly responsive to troubleshooting technical issues. Moreover, as change is the only constant with technology vendors, multiyear studies must be agnostic and agile in building for, using, and adapting to multiple platforms over time.

To this end, the inflammatory bowel disease Specialty Medical Home trial included contingency plans at all sites to use a video platform outside the EMR as a backup, a flexibility facilitated by COVID-19-related reduction in HIPAA regulatory requirements. Furthermore, HomePal began their study using two different video platforms across providers and sites with the knowledge that they would be replaced with another enterprise-wide platform about a year later. Prepping providers and patients ahead of time about that possibility is important and allowed for setting expectations regarding additional training and limited attachment to one platform. This is true for both clinic-to-clinic and clinic-to-home settings, especially the latter, where patients do not have the same access to IT support as they would in a clinic.

## Regulatory and Reimbursement Navigation

### Overview

Telemedicine studies may face fewer regulatory and reimbursement issues in integrated systems of care (ie, the Veteran’s Administration and Kaiser Permanente) as distant and host sites are in the same health care system. All patients are enrolled in the same EMR, and no additional credentialing, privileging, or insurance paneling of telemedicine providers is required. Credentialing is the process of assessing and confirming the license or certification, education, training, and other qualifications of a licensed or certified health care practitioner), and privileging is the process of authorizing a health care practitioner’s specific scope and content of patient care services [15].

In nonintegrated systems of care, scheduling and delivering health care via interactive video can be approached through enrollment in the health care system of the distant site, which requires no additional credentialing, privileging, or insurance paneling for providers. Billing for telemedicine encounters is efficient when done at scale, but providers do not have direct access to EMRs at the host site, increasing the potential for duplicative services (eg, medical tests), over-or contraindicated-prescribing, and lack of care and research coordination. Patients might also face unfamiliar health care and scheduling systems, as well as cost-sharing requirements. A more integrated, patient-centered, and potentially safer approach is to have distant site providers credentialed and privileged to practice, chart, and bill from the host site [16].

### Billing and Remuneration

Medicare coverage analysis is typically performed to determine which expenses are considered *study-related* and which expenses are considered *standard of care*. For billing of study-related expenses, setting up a protocol for invoicing to the prime institution that includes payment terms, timelines, and an invoice template that reflects the terms of the executed agreement will ensure there are no gaps in research activities as a result of billing or payment issues. If a Medicare coverage analysis determines that telemedicine service is the standard of care, then the Centers for Medicare & Medicaid Services regulations and payer procedures will dictate how telemedicine services are reimbursed as a standard of care as a function of state [17].

### Contracting and Budgeting (Indemnity)

The prime institution is responsible for working with participating sites to establish subcontracts. Executing a subaward involves negotiation of terms and fees, which often vary according to each site’s local laws and institutional policies. The scope of work and proposed budget should be prepared ahead of time to allow participating sites to make revisions based on current institutional rates, anticipated personnel effort, and applicable fees for licensing and credentialing. Valuable time may be saved during contract execution if the budget has been completed and reviewed by the subrecipient before the negotiations. Investigators with the Improving Specialty-Care Delivery for Chronic Skin Diseases study engaged with performance sites at the beginning of the proposal development timeline to establish working relationships with the relevant administration and understand institutional contracting procedures and policies. The primary study team was able to receive approval for the financial aspects of all subrecipient efforts before the notice of the award. With these subawards preapproved and key entities from each institution actively engaged, project researchers saved valuable time and were able to begin study conduct at the satellite sites as much as 8 weeks sooner than if budgetary considerations had not already been finalized.

Malpractice is a concern for telemedicine encounters because of the lack of legal precedents. If the distant site specifies the host site as an approved *site of practice*, it will ensure that their distant site’s indemnification coverage extends to the provider. However, the distant site’s malpractice insurance will not cover the host site if named in a lawsuit; the host site will need to purchase supplementary gap indemnity coverage for telemedicine encounters [16].

### Institutional Review Board

The National Institutes of Health policy number NOT-OD-16-094 advises that multisite trials use a single institutional review board (SIRB) to conduct and oversee the protection of human subjects (US HSS, 2016) in an effort to streamline the ethical review process and reduce administrative burden and redundancies inherent to multiple review boards. Although SIRB use may increase the speed of protocol approval, the policy introduces challenges, including the selection of an appropriate institutional review board of record and agreement from institutions to cede review to an outside review board [18].

Use of an SIRB system requires strict adherence to the approved trial protocol from participating sites to ensure the safety of subjects and scientific rigor across the study. Communication and reporting mechanisms for adverse events or unanticipated problems, protocol deviations, and study progress should be established before the initiation of the study. In addition, a standard operating procedure for the storage and maintenance of regulatory documents should be developed and distributed to participating sites to ensure that the sites agree to follow. The coordinating site for reporting and maintenance of regulatory items should establish clear communication and reporting expectations from the outset for the safety and integrity of the trial. The Improving Specialty-Care Delivery for Chronic Skin Diseases trial study team used an SIRB (the University of Southern California Institutional Review Board) to oversee the clinical trial conduct and monitor participant safety across participating sites (University of Colorado and University of California, Davis). Therefore, amendments to procedures and study documents, protocol deviations, and reportable events were submitted to and reviewed exclusively by the University of Southern California Institutional Review Board. For this study, using an SIRB for the multisite trial enabled a streamlined, consistent, and efficient process for oversight of study conduct, patient safety, and enrollment details. In this model, designated individuals from the University of Southern California coordinating team established frequent communication procedures before conducting research. These procedures included weekly team meetings, regular email updates, and a mechanism for requesting ad hoc meetings in the event of a time-sensitive item requiring urgent principal investigator input. The primary site developed and coordinated training activities for all performance site personnel on the institutional review board–approved research protocol and distributed detailed standard operating procedure documents in advance of study activation at each site. Site staff at the primary site were also available for questions and supplemental training, as needed. Frequent and regular communication across sites and standardized and contemporaneous training materials contributed to the well-being of enrolled participants, as study-wide compliance to the research protocol was indicated by the lack of major protocol deviations over the course of the study.

## Design and Initiation

### Overview

Constantly evolving technology, regulations, and payments create significant challenges in the rigor and selection of the study design (ie, parallel control or stepped wedge, quasi-experimental, cluster, or individual-level randomization) and conduct of any comparative effectiveness telehealth study, as these decisions can have substantial implications for implementation success within most complex, adaptive systems [19,20]. Several study design issues should be considered when implementing large, multisite trials involving telemedicine.

### Comparators and Outcome Measurements

*Usual care* and other comparators change over time as telemedicine becomes more mainstream, making it harder to maintain the comparison. It should be noted that significant changes in the delivery of telemedicine occurred during the recent pandemic that likely impacted these types of clinical trials. However, it is too early to objectively assess the extent of the impact and how it will affect these and future trials. Furthermore, understanding a study’s outcome measures and how the data can be collected pragmatically while making use of EMR data is important. The SUMMIT trial will conduct a thorough process evaluation to determine relevant barriers and facilitators of telemedicine-delivered sessions from a multistakeholder perspective, including study participants, identified significant others, treatment providers, and other clinicians. This evaluation will answer key digital technology questions in mental health care [21], including the risks and benefits of using telemedicine versus in-person treatment for mental health care needs. The inflammatory bowel disease Specialty Medical Home trial carefully tracks fidelity to face-to-face versus telemedicine care delivery and records reasons for any cross-contamination observed. Regular meetings are conducted with clinical and research teams, as well as the study statistician, across the three sites and these are also monitored by the stakeholder advisory board to minimize cross-contamination.

### Cross-contamination

In designing multisite trials, it is important to consider the prevention of cross-contamination between arms when examining the efficacy of telemedicine-delivered care compared with another arms. This aspect is closely tied to the study design (eg, randomization at the site, provider, or individual patient level). Developing implementation rules, involving key stakeholders in development, and physically separating implementation platforms can be useful in avoiding cross-contamination. Sharing clear rules of telemedicine-based arms with key stakeholders is essential, including study participants during informed consent, treatment providers during training, and others who may prefer one arm versus another (eg, referring clinicians). Arms can also be separated by training one cadre of providers to use the telemedicine-based platform, whereas the other uses the alternative. Finally, including key stakeholders in study development provides the opportunity to identify relevant barriers [22] and ensures a more holistic and patient-centered approach. The C3FIT study was randomized by clinical site and went to great lengths to keep their two study arms separated, appointing different personnel to work exclusively with each arm, holding separate arm-specific meetings, and sending separate communication, including tailored versions of monthly newsletters and other correspondence. It is also important to acknowledge that researchers cannot control every aspect of a study, including technology. For example, during the COVID pandemic, non–HIPAA-compliant technologies were allowed, and even though a study might require a specific platform, some providers might have chosen to use a different platform because it was more expedient, which is a potential source of cross-contamination.

### Telemedicine Platform Training Protocol

Developing a training protocol is critical for ensuring quality patient care and meaningful outcome data. Providers and site staff should be trained accordingly, as additional participating sites will likely introduce increased variability in the use of the platforms. To ensure standardization, a training protocol that includes a detailed, step-by-step description of how and when to use the platform should be developed. In-person training sessions, video tutorials, and informational sheets are helpful resources to provide participating site personnel to ensure consistent adherence to the telemedicine platform procedures. C3FIT included a combination of recorded video training using a web-based training platform combined with short, focused, and ongoing web-based meetings with site personnel to reinforce training and have continued to reinforce these messages as the trial commenced. Role playing and superuser testimonials and tips were helpful components of HomePal’s web training sessions. In the Improving Specialty-Care Delivery in Chronic Skin Diseases study, the main training and procedural components mandated for each site before activation were completion of a tutorial use of the selected telemedicine platform. Research coordinators, investigators, and providers were provided with a standardized training session customized to their role in the study and their access within the platform. For example, research coordinators were trained specifically on registration of patients to the web-based system, instructions for patients on how to use the platform to submit cases, and use of scheduling and messaging functionality of the system. Training for dermatologists and primary care physicians focused primarily on how to review patient cases, submit recommendations and prescriptions, and communicate securely with the patients, other providers, and the study team via the web-based platform. Training materials developed by the coordinating center were available for review by participating site personnel at any time to ensure consistent adherence to the telemedicine platform procedures.

## Health Disparities

The fundamental aim of telemedicine is to increase access to care [23]. However, as with any new technology, adoption is often not equitable, and considering the impact on disparate populations is essential. Contributors to disparities [24] include the global divide between high- and low-income societies, a social divide between the information-rich and information-poor, and a democratic divide between those who use digital tools to engage in public life and those who do not. The potential for telemedicine initiatives to improve health care access [23] and reduce health disparities has been widely noted [25-28], particularly given ongoing physician and specialty shortages in rural areas. Telemedicine offers an almost unprecedented opportunity to break down geospatial barriers to care and expand access for underserved populations. Likewise, well-designed multisite trials have the potential to identify, refine, and provide evidence on how telemedicine can be used to reduce socioeconomic, racial, ethnic, and geographic disparities in care.

Although great promise exists, disparities in access to care continue and are driven by complex issues that often require broad infrastructure investments to correct [27,29]. Rural and underserved areas considering telemedicine implementation face high equipment and start-up costs, limited personnel and reimbursement, and interoperability issues that limit collaboration [27,29], as well as policy and regulation restrictions [27]. Furthermore, inadequate access to broadband, particularly among rural residents, can limit access to telemedicine services [30], as can sociodemographic differences [27].

Since its beginning, telemedicine has sought to bridge these divides, but researchers must be conscious of these inequities and make efforts to not perpetuate them in the design and conduct of their studies. Multisite telemedicine intervention trials may maintain or increase existing disparities rather than decrease disparities for a number of reasons, as described below:

1. The telemedicine program or intervention must be designed for clinics, hospitals, or institutions that serve the most disadvantaged populations. If clinical sites can more easily use and implement the program or intervention, there is an increased likelihood of a stronger health impact for disadvantaged populations. For example, if limited English proficiency patients and families cannot be accommodated during telemedicine visits, the potential impact may be limited to an important and often disadvantaged population [31]. In the Telehealth-Co-ordinated Referral project, researchers brought a federally qualified health center, local community mental health clinics, and parents together to design and implement an intervention to fit the resources, capacity, and needs of its stakeholders (primary care clinicians, specialty mental health care clinicians, and parents) [32].
2. The program or intervention must be acceptable to its users, including providers, staff, patients, and families. For example, providers who care for more underserved patients often work in clinical facilities that are less resourced [33]. If these providers find the intervention too cumbersome for participation because of limited resources (eg, lack of sufficient nursing staff), they may be less likely to fully engage, thus altering the potential impact on the most disadvantaged patients. Another example is direct-to-consumer (DTC) telemedicine, which should be accessible preferentially by patients in more rural areas and in areas designated as Health Resources and Services Administration and primary health care professional shortage areas; however, a recent study of DTC demonstrated that users of one DTC service were not preferentially located in rural or primary health care professional shortage areas [34].
3. The study protocol must be designed with the most disadvantaged group. For example, if the study protocol requires patients to complete a lengthy written survey, patients with lower literacy levels may be less likely to complete it; thus, the impact on these patients will not be well understood [35]. If the study requires patients to provide a social security number to receive a study incentive, undocumented immigrant patients may decline enrollment because of fear of immigration enforcement [36-38].

Connect.Parkinson, one of the first national randomized controlled trials to assess the feasibility, value, and benefits of specialist-led telemedicine visits for Parkinson disease, demonstrates the ability of telemedicine to address geographic barriers to care. Participants from roughly 20 US states, many in remote locations, participated in >90% of the visits completed. Each visit saved patients and their caregivers about 100 miles of travel and 3 hours of time. Thousands expressed interest in this telemedicine study, and the care model, assessed in 2014, foresaw the large latent demand and high receptivity for telemedicine among many older adults that emerged during the COVID-19 pandemic [39,40]. A limitation of this trial was that although it did address geographic disparities, it did not address social disparities, as the majority of participants were White and college educated. However, given the positive outcomes for these patients with Parkinson disease, this trial could readily be translated to more disadvantaged populations.

Researchers engaging in multisite telemedicine trials can do several things to enhance their study’s ability to reduce health disparities:

1. Understand the underlying pre-existing health and health care disparities that relate to telemedicine intervention under study in the trial.
2. Incorporation of health systems, providers, staff, and patients that represent the most disadvantaged groups as partners and collaborators in the study design, implementation, planning, and conduct.
3. Design the trial to collect reliable demographic data on race and ethnicity, language, income, and rurality (at least) for all participants.
4. Power the study so that intervention impact can be studied by participant demographic factors (ie, race and ethnicity, and language). This may require certain populations to be overrepresented in their recruitment strategy.

HomePal addressed disparities in digital access and literacy by using *telepresenters* in the form of nurses who were at home to facilitate a remote video consultation with physicians. These nurses could work out any technology and clinical issues, which lessened the impact on patients and caregivers, which is particularly important for those without a supportive home environment. Furthermore, C3FIT sought to decrease access and financial barriers when COVID-19 prohibited in-person visits by providing internet-enabled tablets to participants who did not have access to a smartphone or computer to conduct study visits. The Connect.Parkinson trial mailed a webcam to participants who did not have one, and the SUMMIT study provided tablets to participants lacking access to technology.

## Implementation and Sustainability

### Overview

Sustainability may differ depending on the specific intervention being evaluated (eg, telemonitoring, telediagnostics, video consultations, and telecounseling). Implementation planning will benefit from using several frameworks to assess potential facilitators and barriers [41-43], readiness for large-scale trials [44], and adjustment of study design or approach to maximize pragmatism in real-world settings [45].

### Ensuring Health System Leaders, Providers, and Support Staff Buy-in

Pearl [46] identified five key areas to improve physician engagement in telehealth that readily apply to getting buy-in for research projects:

1. Communicate the *Why:* Understanding the research and patient value, including the convenience of receiving medical care without travel or missing school and work, tends to resonate with physicians and other personnel. HomePal physicians were sold on using video visits and saw improved efficiency in seeing patients and reductions in windshield time; however, this benefit was not seen by home-based nurses, who managed both technology and synching up with physician schedule and availability [47].
2. Compensate appropriately: compensating physicians based on relative value units or production incentives can be incentives for the adoption of telehealth and engagement in research, but including them as coinvestigators on grants and authors on publications can incentivize participation as well. Setting performance targets and incentives may assist by motivating the competition.
3. Keep it simple: Cumbersome and flawed IT that requires multiple applications and clicks to move through will impede physician adoption and potentially impact data quality.
4. Mind the workload: In general, telemedicine visits require the same time commitment as in-person visits; the provider should be given adequate support and staff to encourage its use and to carry out any additional research tasks.
5. Invest in the culture: a shared EMR and technology and ensuring that rather than being penalized, providers will be encouraged to use telemedicine innovations will improve adoption and research engagement.

### Patients and Family Caregiver Engagement

To optimize the engagement and representativeness of the study sample, designs must allow the care team to frame and set expectations that telemedicine tools will be used as appropriate for routine care to best meet the needs of patients and families. This approach allows for tailoring the technology to the needs and preferences of patients and families and the clinical situation, which ultimately reflects real-world practices. Video visits may not be appropriate for many older patients with hearing or vision impairment; however, video facilitates lip reading and is considered superior to phone if transportation to a clinic is not possible. Furthermore, patients and families must be assured that they can still easily access clinic visits or have a clinician to make a home visit if needed. Using home health staff to facilitate video consults with providers may overcome access disparities for the most vulnerable homebound populations [48,49]. This was especially important for HomePal, as their physicians could still make home visits if the video consults suggested a need to do so, which led to increased assurance by the patient and family regarding quality of care. Using video allowed multiple family caregivers to join the session if needed to ensure a care plan agreement.

### Sustainability

Positive developments with telehealth regulations and payments in the United States have set the stage for telehealth to be an integral tool for care delivery. Thus, research questions will focus less on whether telehealth tools are effective or as good as the in-person gold standard, and more on how specific tools can be best leveraged, under what conditions to enhance quality of care and outcomes as systems and regulations evolve [50]; the telehealth intervention tested at the start of the study may not be the same at the end of the study. The pragmatic study design helps, in general, to foster sustainability after the study is completed.

### Leadership and Funding

The importance of strong leadership and adequate funding in the development and implementation of successful telemedicine trials is especially important and cannot be understated. Telemedicine and telehealth service research has stressed the importance of identifying *champions* who can promote uptake, legitimize services, build relationships, and work through implementation challenges [20,51]. Having champions when building and executing telemedicine trials is essential, not only at the lead site or sites but also at clinical sites to allow working through institution-specific processes and barriers.

Although understanding the specific resources required to support telemedicine trials is still evolving, these types of trials are often expensive and require more resources than traditional trials. To obtaining sufficient funding, leaders who can advocate for and incorporate creative approaches to support may frequently be necessary. One question is whether to pay for clinical services out of a grant. Although possible, this should be used with caution to avoid creating potential bias in the trial (ie, including only insured patients). As with traditional clinical trials, every effort should be made to include as many patient-related expenses in the grant budget as much as possible. It is important to include telemedicine-specific items such as devices (eg, smartphones, tablets, electronic scales, blue-tooth-enabled blood pressure monitors), as in the C3FIT study. In many trials, these can be cycled through different patients as each patient completes the trial.

In many cases, grant funding alone is not adequate to cover all trial expenses. Researchers need to be creative at times to cover these expenses. For example, there are a number of companies with telemedicine products and platforms that are willing to collaborate on trials sometimes providing technology for free or at reduced prices with the hope that they will be included in future clinical budgets if they perform well. There are also state and federal programs designed to help providers and patients with telemedicine technology support. For example, during the COVID-19 pandemic the Federal Communications Commission had at least two rounds of funding, with one having dedicated funds to help patients with device and internet access.

## Discussion

This manuscript provides a blueprint of lessons learned from seven PCORI trials in various stages of development and design through training and implementation, as well as the impact on disparities. A feasible approach for telemedicine implementation is a necessary part of any telemedicine research study, and must be integrated into the study protocol and timeline, and this blueprint represents perspectives and provides potential solutions from researchers across the continuum, from just beginning their research to further in the process of completing multisite telemedicine trials.

Consumers, major drivers of telemedicine use, point to improved convenience and access to care as important benefits. Research has found high numbers of consumers who rate the convenience and access improvements important and are willing to try telemedicine visits [52], as well as extremely high levels of satisfaction [53]. This suggests that use of telemedicine and its use in multisite trials will only increase if designed and implemented successfully.

Minority and low-income populations have difficulty accessing care because of age, health, social support, location of medical centers, and numerous other social issues. There must be a greater positive impact on the most disparate or most disadvantaged group [54-56]. If the intervention works equally well in all groups or populations, disparity will be maintained. If the intervention works better in the more advantaged group, the disparity will widen. Without this type of blueprint for multisite telemedicine trials, we may miss the opportunity to create and implement telemedicine programs that actually improve existing disparities.

The populations that telemedicine serves will also expand from episodic to chronic conditions. Motivation for use will also expand from convenience to access to better patient-centered care. Finally, telemedicine applications will expand from hospitals and clinics to home and mobile devices [57]. As this migration occurs, many patients will require the support of local clinicians, such as those in community-based outpatient clinics that support millions of veterans [58]. These changes, combined with increasing access to the internet and familiarity with its health applications, will enable larger and more representative populations to participate in research. The challenge will remain to meet these individuals on their terms and to support them with services that are tailored to their needs.

## Abbreviations

C3FIT: Co-ordinated, Collaborative, Comprehensive, Family-based, Integrated, Technology-enabled Care
DTC: direct-to-consumer
EMR: electronic medical record
HIPAA: Health Insurance Portability and Accountability Act
IT: information technology
PCORI: Patient-Centered Outcomes Research Institute
SIRB: single institutional review board
SUMMIT: Scaling Up Maternal Mental Healthcare by Increasing Access to Treatments
